# A tale of two seas: contrasting patterns of population structure in the small-spotted catshark across Europe

**DOI:** 10.1098/rsos.140175

**Published:** 2014-11-12

**Authors:** Chrysoula Gubili, David W. Sims, Ana Veríssimo, Paolo Domenici, Jim Ellis, Panagiotis Grigoriou, Andrew F. Johnson, Matthew McHugh, Francis Neat, Andrea Satta, Giuseppe Scarcella, Bárbara Serra-Pereira, Alen Soldo, Martin J. Genner, Andrew M. Griffiths

**Affiliations:** 1 School of Environment and Life Sciences, University of Salford, Salford, Greater Manchester M5 4WU, UK; 2 Marine Biological Association of the United Kingdom, The Laboratory, Plymouth PL1 2PB, UK; 3 CIBIO-U.P., Centro de Investigação em Biodiversidade e Recursos Genéticos, Campus Agrário de Vairão, Rua Padre Armando Quintas, Vairão 4485-661, Portugal; 4 CNR-IAMC Località Sa Mardini, Torregrande 09170, Italy; 5 Centre for Environment, Fisheries and Aquaclture Science (CEFAS), Pakefield Road, Lowestoft, Suffolk NR33 0HT, UK; 6 Cretaquarium, Thalassocosmos, Hellenic Centre for Marine Research (HCMR), PO Box 2214, Heraklion Crete 71003, Greece; 7 Center for Marine Biodiversity and Conservation, Scripps Institution of Oceanography 0202, University of California, 9500 Gilman Drive, San Diego, CA 92083-0202, USA; 8 Marine and Estuarine Ecology Unit, School of Biological Sciences, University of Queensland, Brisbane, Queensland 4072, Australia; 9 Marine Scotland—Science, Marine Laboratory, PO Box 101, Aberdeen AB11 9DB, UK; 10 ISMAR-CNR—Istituto di Scienze Marine, Consiglio Nazionale delle Ricerche, Largo Fiera della Pesca 2, Ancona 60125, Italy; 11 Departamento do Mar e Recursos Marinhos, IPMA, Instituto Português do Mar e da Atmosfera, Av. Brasilia, Lisboa 1449-006, Portugal; 12 Department of Marine Studies, University of Split, Livanjska 5, Split 21000, Croatia; 13 School of Biological Sciences, University of Bristol, Bristol Life Sciences Building, 24 Tyndall Avenue, Bristol BS8 1TQ, UK

**Keywords:** elasmobranch, sex-biased dispersal, fisheries management, Scyliorhinidae, lesser spotted dogfish

## Abstract

Elasmobranchs represent important components of marine ecosystems, but they can be vulnerable to overexploitation. This has driven investigations into the population genetic structure of large-bodied pelagic sharks, but relatively little is known of population structure in smaller demersal taxa, which are perhaps more representative of the biodiversity of the group. This study explores spatial population genetic structure of the small-spotted catshark (*Scyliorhinus canicula*), across European seas. The results show significant genetic differences among most of the Mediterranean sample collections, but no significant structure among Atlantic shelf areas. The data suggest the Mediterranean populations are likely to have persisted in a stable and structured environment during Pleistocene sea-level changes. Conversely, the Northeast Atlantic populations would have experienced major changes in habitat availability during glacial cycles, driving patterns of population reduction and expansion. The data also provide evidence of male-biased dispersal and female philopatry over large spatial scales, implying complex sex-determined differences in the behaviour of elasmobranchs. On the basis of this evidence, we suggest that patterns of connectivity are determined by trends of past habitat stability that provides opportunity for local adaptation in species exhibiting philopatric behaviour, implying that resilience of populations to fisheries and other stressors may differ across the range of species.

## 2. Introduction

Molecular genetic markers have had a profound impact in conservation and management [1–3]. They allow inferences to be made about the scale over which genetic connectivity occurs [4], the behavioural mechanisms leading to gene flow [5], the estimation of effective population sizes [6] and how historical processes have impacted on populations [7]. This information has importance in terms of potential for local adaptation [8,9], but is also valuable for sustainable management and conservation of these natural resources [10–13]. In fisheries management, genetic tools can help to identify discrete populations which represent demographically independent stocks that require individual management to ensure fisheries sustainability [10,14].

The effects of marine overexploitation have been clearly shown by severe declines in many shark species [15]. Understandably, much of the work investigating genetic diversity and population structure of elasmobranchs has been focused on the large pelagic sharks that are considered particularly vulnerable to exploitation in high seas fisheries [16,17]. However, these species represent a small fraction of the biodiversity of the group. The small-spotted catshark (*Scyliorhinus canicula* L., 1758) is a relatively small demersal species belonging to one of the largest families of sharks, the Scyliorhinidae. It is generally considered to be the most abundant catshark in European shelf seas [18] and occurs from Norway and the British Isles, south to Senegal, including the Mediterranean Sea [19]. It is an oviparous species that breeds most of the year and is relatively fecund for an elasmobranch. It has remarkable variation in reproductive parameters, but in British waters it has been shown to lay between 29 and 62 eggs from November to July each year [18,20]. In the Atlantic, it is often caught as by-catch in demersal fisheries, but its commercial importance is growing, particularly through its use as whelk bait [21], and it is also significant for recreational fishing in some regions [22]. In the Mediterranean, catsharks have been fished since ancient times, as documented by mosaics from the Roman age [23], and *S. canicula* is still targeted for consumption today [24]. Recent studies have shown very dramatic localized reductions in abundance [25]. For example, in the Adriatic Sea it has been estimated that the species has declined in abundance by up to 90% since the 1940s [26].

Investigations of elasmobranch population structure focusing on wide-ranging pelagic sharks have often revealed genetic differentiation over broad inter- or intra-oceanic scale [27,28]. By contrast, work on coastal and demersal species suggests they can have more highly divided population structure, which has implications for management and conservation [29,30]. *Scyliorhinus canicula* has a range of traits associated with a low dispersal potential, including internal fertilization and deposition of demersal eggs. In addition, mark–recapture studies suggest adults do not generally make long migrations [31]. These factors could potentially lead to population genetic structure in this species, a concept that has some support from apparent differences among populations in growth rates, habitat/depth preference and reproductive biology that could have arisen from local adaptation [20,25]. Indeed, populations within the Mediterranean show such marked changes from those in the Atlantic that they have historically been suggested as a different subspecies [32,33]. Similarly, a more recent study of sexual dimorphism in *S. canicula* noted significant morphological differences in dentition between west African, Mediterranean and west European populations, indicating the west African group could represent a distinct taxon [34].

Documented sex-biased dispersal and philopatry have also been shown to have serious effects on elasmobranch population structure. Female sharks generally make far greater investment in reproduction than males, potentially leading to discrepancies between optimal male- and female-fitness strategies, and generating sex-specific differences in behaviour [27,35]. Evidence from a growing number of studies suggests female philopatry in sharks may be widespread [36]. This is also significant as sex-biased differences in movement behaviour could potentially lead to sexual segregation in sharks, and in turn affect the sustainability of marine harvesting [37].

Molecular data have the power to infer historic processes, such as population expansions or contractions, locations of refugia and patterns of recolonization [38]. Inferences of this type are particularly enlightening within Northeast Atlantic marine ecosystems, as organisms in this region have been significantly impacted by the Pleistocene glacial cycles [39]. During the last glacial maximum, about 20 000 years ago, ice sheets dominated the majority of the United Kingdom and Ireland, while permanent sea ice may have extended as far south as the Bay of Biscay [40,41]. Therefore, the distributions of marine organisms might have been forced southwards into refugia, including the Mediterranean, north African coast and the Iberian Peninsula [42–44]. Along the Atlantic coast, there is also evidence for refugia further north and much closer to the ice sheets [43,45,46]. Subsequently, as the ice retreated, organisms were able to recolonize the more northerly regions that were previously glaciated. Phylogeographic investigations of a variety of marine taxa have shown a division between the Mediterranean Sea and the Atlantic, although the degree and geographical scale of the biogeographic separation varies among even closely related species [47]. Therefore, it is plausible that these locations also acted as refugia for the small-spotted catshark.

Here, we test for population genetic structure among populations of *S. canicula* collected across European seas. A particular focus is made of the Atlantic–Mediterranean transition, as it is often considered to be an important phylogeographic break. We also use these data to test for sex-biased differences in dispersal and philopatry. We discuss the results in the light of published work on the behavioural ecology of the species, and highlight the conservation and management implications.

## 3. Material and methods

### 3.1 Sample collection and DNA extraction

**Figure 1.**
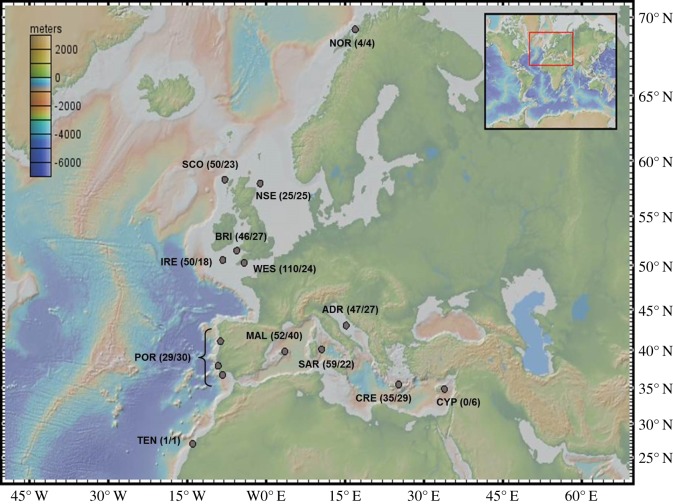
Sample collection locations, with numbers of individuals analysed (microsatellite/mitochondrial markers). Inset colour scale reflects sea depth. See table 1 for location codes.

Tissue samples were collected between 2007 and 2011 (with the exception of the Western English Channel collection site, see below), primarily from research cruises throughout Europe and the Mediterranean (figure 1; electronic supplementary material, S1). Temporally replicated samples were collected from the same approximate region of the Western Channel (2003, *n*=45; 2008, *n*=26; 2010, *n*=39). A minority of samples were also collected at landing or at fish markets, most notably all those from Portugal, Sardinia and Crete. Genomic DNA was isolated from *S. canicula* using the Wizard technique (Promega Madison, WI, USA).

### 3.2 Microsatellite genotyping

Twelve microsatellite loci, Scan02, Scan03, Scan04, Scan05, Scan06, Scan09, Scan10, Scan12, Scan13, Scan14, Scan15 and Scan16 [48], were amplified with the QIAGEN multiplex polymerase chain reaction (PCR) kit (QIAGEN, Valencia, CA, USA). The protocol followed Griffiths *et al*. [21], with the following modifications. Fluorescent NED primers were used to ensure compatibility with ABI sequencing platforms, and loci Scan01 and Scan17 were removed from the original multiplex owing to problems associated with null alleles and PCR artefacts (with Scan04 and Scan10 substituted instead). Allele sizes were determined using an ABI3500 DNA sequencer, and the STRand nucleic acid analysis software [49].

### 3.3 Mitochondrial DNA sequencing

An approximately 900 base pair (bp) section of the mitochondrial DNA (mtDNA) control region was amplified using the newly designed primers ScyD1pF (ATGACATGGCCCACATATCC) and Scan2R (TTCTCTTCTCAAGACCGGGTA), using PCR conditions described in Griffiths *et al*. [50]. PCR products were cleaned and sequenced by Macrogen (Korea), using the forward primer ScyD1pF, which yielded a shorter region that was used for subsequent analyses. Resulting sequences were checked using BioEdit v. 7.0.9 [51] and aligned with ClustalX2 [52].

### 3.4 Microsatellite data: summary statistics and population differentiation

Patterns of temporal variation at the Western Channel site were examined by calculating pairwise *F*_ST_ values between the sampling periods in Arlequin 3.11 [53]. Microchecker [54] was used to check for scoring issues and the presence of null alleles. Microsatellite summary statistics were calculated in Cervus 3.0 [55], except the inbreeding coefficient (*F*_IS_), probability of conformity to the expectations of Hardy–Weinberg equilibrium (HWE) and linkage disequilibrium (LD) which were calculated in Genepop 4.0.7 [56]. Allelic richness was also estimated in FSTAT 2.9.3.2 [57].

Sample collections with inappropriately small sample sizes for estimating allelic frequencies from microsatellite loci (i.e. from Tenerife *n*=1 and Norway *n*=4; figure 1) were removed. Estimation of pairwise *F*_ST_ values was conducted in Arlequin using 10 000 randomizations, and 95% CI were estimated across loci in GDA 1.0 [58] with 10 000 bootstraps. Sequential Bonferroni corrections were used to minimize type I errors [59]. Within GenAlEx 6.5 [60], Nei’s pairwise genetic distances were calculated using the default settings and visualized by principal coordinate analysis (PCoA). Mantel tests were used to test for significant correlations between genetic distances and geographical distances, also in GenAlEx.

A hierarchical analysis of molecular variance (AMOVA) was performed in Arlequin, to test for significance between groups of sample collections. Data were grouped according to geographical location, with the following hierarchy: Northeast Atlantic (Scotland, North Sea, Bristol Channel, Western Channel, Ireland and Portugal) and the Mediterranean (Mallorca, Sardinia, the Adriatic and Crete).

### 3.5 Microsatellite data: clustering analysis

The complete microsatellite dataset was analysed in the software Structure v. 2.3.4 [61]. A ‘hierarchical’ approach [62] with multiple rounds of analysis was employed in order to capture the major structure within the data. Run-lengths included a 100 000 burn-in and 1 000 000 total length, with five iterations. The model incorporating sampling locations as prior information was employed and the numbers of clusters, *K*, varied between 1 and 12 in each run. The *ΔK* method of Evanno *et al*. [63] was applied to judge the most likely values of *K*. In the analysis of each cluster, plots of the absolute values of ln Pr(*X*|*K*) and *ΔK* were generated by Structure Harvester [64].

### 3.6 Mitochondrial DNA: summary statistics and population differentiation

Summary statistics were primarily calculated using Arlequin. Rarefied haplotype richness, standardized to a sample size of 22, was calculated using Contrib 1.02 [2]. Genetic differences among localities were estimated in Arlequin, using both the genetic distance-based *Φ*_ST_ and the frequency-based mt*F*_ST_, with significance of differences estimated with 10 000 permutations. A hierarchical AMOVA was performed in Arlequin, using the same scenarios as implemented with the nuclear markers. Tamura and Nei’s [65] genetic distances were calculated between all sample collections (excepting the lone African/Canaries sample) using MEGA v. 5.1 [66] and visualized using PCoA in GenAlEx. Mantel tests were used to look for significant correlations between genetic distances and geographical distances, also in GenAlEx.

### 3.7 Mitochondrial DNA: haplotype network and demographic analysis

A median joining network was used to investigate genealogical relationships between mtDNA haplotypes with Network 4.6.1.1 [67]. Tajima’s *D* [68] and Fu’s *F*_*s*_ [69] tests were calculated in Arlequin [53], with significant negative values indicative of recent population expansion. The demographic history was also evaluated by mismatch distribution analysis. Typically, a population of constant size is characterized by a multimodal distribution; alternatively, one that has experienced expansion usually shows a unimodal distribution [70]. Bayesian skyline plots (BSPs) were generated in the software package BEAST v. 1.7 [71], and plotted using the upper 95% highest posterior density. Generally the programme defaults were used, except the *HKY*+*Γ*+*I* mutation model was selected and the Markov chain Monte Carlo (MCMC) was set between 50 and 200 million iterations, depending on length required for convergence. A fixed clock was set using a divergence rate of 0.361% estimated from homologous control sequences from *S. canicula* and *Scyliorhinus stellaris* (AM Griffiths 2010, unpublished data) and the divergence date (22 Ma) from Sorenson *et al*. [72]. This is the closest calibration point available, but it has resulted in a comparatively slow estimate rate of divergence, albeit one broadly similar to those calculated for mtDNA in other sharks (0.8% [73], 0.67% [74]). It is important to highlight the uncertainty in estimation of the clock rates, which could have very significant effects on the phylogeographic reconstructions in the BSP.

### 3.8 Sex-biased dispersal

Initially, to assess differences between male and female dispersal a test comparing pairwise genetic distances between populations for microsatellite and mitochondrial data was employed. Specifically, with male-biased dispersal, lower genetic distances were expected to be present in biparentally inherited (microsatellite) markers than in maternally inherited (mitochondrial) markers. Following Daly-Engel *et al*. [35], paired *t*-tests in *R* 2.15.1 were used to compare mt*F*_ST_ calculated using mtDNA haplotype frequencies and *F*_ST_ calculated using microsatellite allele frequencies.

Tests for sex-biased dispersal based on the microsatellite data alone were conducted in FSTAT. Samples from Sardinia were removed from analyses as no information on sex was available. Five methods based on differences on the inbreeding coefficient (*F*_IS_), fixation index (*F*_ST_), degree of relatedness, mean assignment indices (mAIc) and variance of the assignment indices (vAIc) between the philopatric and dispersing groups were used [75]. In principle, unequal levels of gene flow between males and females would lead to a Wahlund effect, and a heterozygote deficit resulting in a higher *F*_IS_ in the most dispersing sex, while also leading to correspondingly lower *F*_ST_ and relatedness values. The assignment index statistic indicates the probability of a genotype occurring in a population, and unequal levels of gene flow between males and females could lead to negative values of the mAIc in the most dispersing sex (as the distribution is centred on zero), while also increasing the corresponding vAIc.

## 4. Results

### 4.1 Microsatellite data: summary statistics and population differentiation

No significant differences were detected between temporal samples from the Western Channel (pairwise *F*_ST_ ranged from less than 0.000 to 0.006 and none were significant at the 99% CI) and all individuals were grouped together into a single sample collection. The mean observed heterozygosity across all populations was 0.632, varying between 0.571 (Mallorca) and 0.659 (North Sea). The mean expected heterozygosity across populations was 0.615, varying between 0.562 (Mallorca) and 0.661 (Portugal; table 1; electronic supplementary material, S2). There was no evidence of deviation from HWE after sequential Bonferroni correction (electronic supplementary material, S2). Furthermore, there was no consistent evidence of scoring issues or null alleles. There were 37 (of 858, 4.3%) significant tests of LD across sample sites at the 95% CI, none of which remained significant after sequential Bonferroni correction. Mean allelic richness across loci varied between 5.320 (Mallorca) and 6.200 (Portugal; table 1). Across all 12 loci, significant genetic differentiation was observed (global *F*_ST_=0.039, *p*<0.001). Pairwise *F*_ST_ values ranged from −0.005 to 0.070 and were significant for all combinations that included the Mediterranean sampling sites, while genetic differentiation was absent among all Northeast Atlantic populations, after Bonferroni correction (table 2).

**Table 1.** Summary statistics across all microsatellite loci. *n*, sample size; *N*_a_, number of alleles; *H*_O_, observed heterozygosity; *H*_E_, expected heterozygosity; *R*_S_, allelic richness; HWE, probability of conformance to Hardy–Weinberg equilibrium. The single sample from Africa/the Canaries is excluded from this table owing to the small sample size. Allelic richness values were calculated after excluding the Norway sample owing to the small sample size, so rarefaction standardized to the level of North Sea collection (*n*=25).

sample collection	code	*n*	*N*_a_	*H*_O_	*H*_E_	*R*_S_	HWE
Norway	NOR	4	3.33	0.6042	0.6161	—	0.9994
Scotland	SCO	50	7.08	0.6025	0.6100	6.02	0.5828
North Sea	NSE	25	5.58	0.6585	0.6250	5.55	0.9803
south Ireland	IRE	50	6.92	0.6165	0.6306	6.00	0.0581
Bristol Channel	BRI	46	6.17	0.6407	0.6311	5.57	0.3087
Western Channel	WES	110	7.67	0.6380	0.6339	5.88	0.1851
Portugal	POR	29	6.42	0.6353	0.6605	6.20	0.0756
Mallorca	MAL	52	6.17	0.5706	0.5620	5.32	0.8064
Sardinia	SAR	59	6.33	0.5793	0.5917	5.45	0.7946
Adriatic	ADR	47	6.08	0.6373	0.6282	5.39	0.0681
Crete	CRE	35	5.92	0.5823	0.5727	5.45	0.6016

**Table 2.** Microsatellite pairwise differentiation among 10 sampling localities. Below diagonal, pairwise *F*_ST_ values (with 95% CI in brackets). Above diagonal, *p*-values. Values in bold were significant at the 95% CI, and those marked with asterisks (***) remained significant after sequential Bonferroni corrections.

locality	Scotland	North Sea	south Ireland	Bristol Channel	Western Channel	Portugal	Mallorca	Sardinia	Adriatic	Crete
Scotland	—	0.024	0.619	0.459	0.267	0.001	<0.001	<0.001	<0.001	<0.001
North Sea	**0.008** (**0.031 to −0.004**)	—	0.462	0.200	0.348	0.632	<0.001	<0.001	<0.001	<0.001
south Ireland	0.000 (0.004 to −0.005)	0.000 (0.009 to −0.007)	—	0.991	1.000	0.095	<0.001	<0.001	<0.001	<0.001
Bristol Channel	0.000 (0.010 to −0.006)	0.002 (0.011 to −0.004)	−0.005 (−0.001 to −0.007)	—	0.995	0.267	<0.001	<0.001	<0.001	<0.001
Western Channel	0.001 (0.010 to −0.003)	0.000 (0.009 to −0.005)	−0.003 (−0.002 to −0.004)	−0.002 (−0.001 to −0.003)	—	0.447	<0.001	<0.001	<0.001	<0.001
Portugal	**0.014** (**0.039–0.000**)	−0.002 (0.008 to −0.009)	0.005 (0.013 to −0.002)	0.002 (0.010 to −0.003)	0.000 (0.007 to −0.003)	—	<0.001	<0.001	<0.001	<0.001
Mallorca	0.043^***^ (**0.065–0.021**)	0.028^***^ (**0.050–0.008**)	0.038^***^ (**0.058–0.020**)	0.038^***^ (**0.056–0.020**)	0.039^***^ (**0.058–0.021**)	0.046^***^ (**0.080–0.018**)	—	<0.001	<0.001	<0.001
Sardinia	0.039^***^ (**0.055–0.026**)	0.038^***^ (**0.065–0.018**)	0.046^***^ (**0.072–0.027**)	0.044^***^ (**0.066–0.027**)	0.050^***^ (**0.059–0.027**)	0.041^***^ (**0.075–0.031**)	0.050^***^ (**0.076–0.026**)	—	<0.001	<0.001
Adriatic	0.043^***^ (**0.063–0.027**)	0.041^***^ (**0.050–0.013**)	0.209^***^ (**0.064–0.022**)	0.039^***^ (**0.062–0.021**)	0.038^***^ (**0.058–0.023**)	0.038^***^ (**0.061–0.020**)	0.064^***^ (**0.085–0.047**)	0.025^***^ (**0.040–0.012**)	—	<0.001
Crete	0.055^***^ (**0.086–0.028**)	0.055^***^ (**0.079–0.032**)	0.057^***^ (**0.086–0.028**)	0.050^***^ (**0.075–0.025**)	0.056^***^ (**0.080–0.028**)	0.059^***^ (**0.102–0.019**)	0.070^***^ (**0.106–0.037**)	0.056^***^ (**0.079–0.032**)	0.039^***^ (**0.064–0.015**)	—

**Figure 2.**
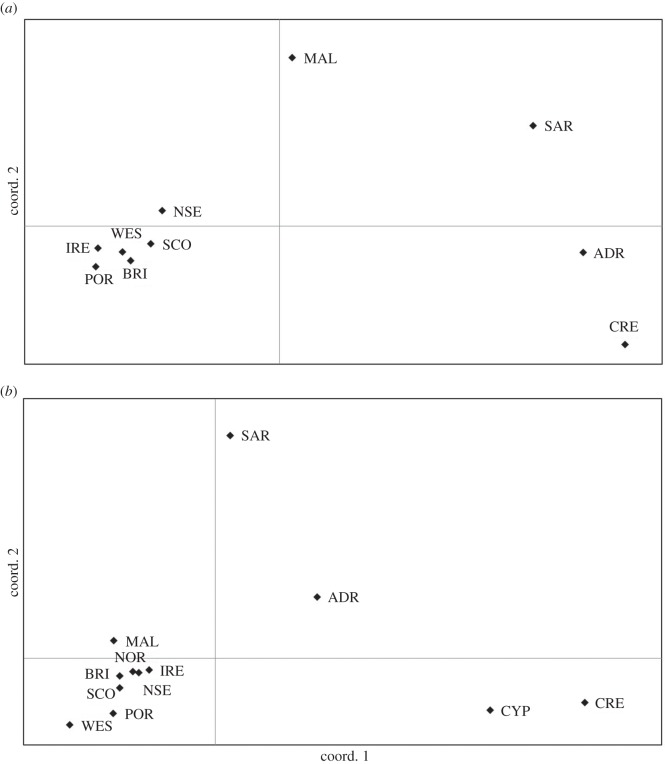
Principal coordinates analysis of (*a*) Nei’s pairwise genetic distances between sample collections based on microsatellite data, where axis 1 explains 39.66% and axis 2 explains 18.78% of the variation in the data, and (*b*) Tamura & Nei’s pairwise genetic distances between sample collections based on mtDNA data, where axis 1 explains 28.22%, and axis 2 explains 13.84% of the variation in the data. See table 1 for location codes.

**Figure 3.**
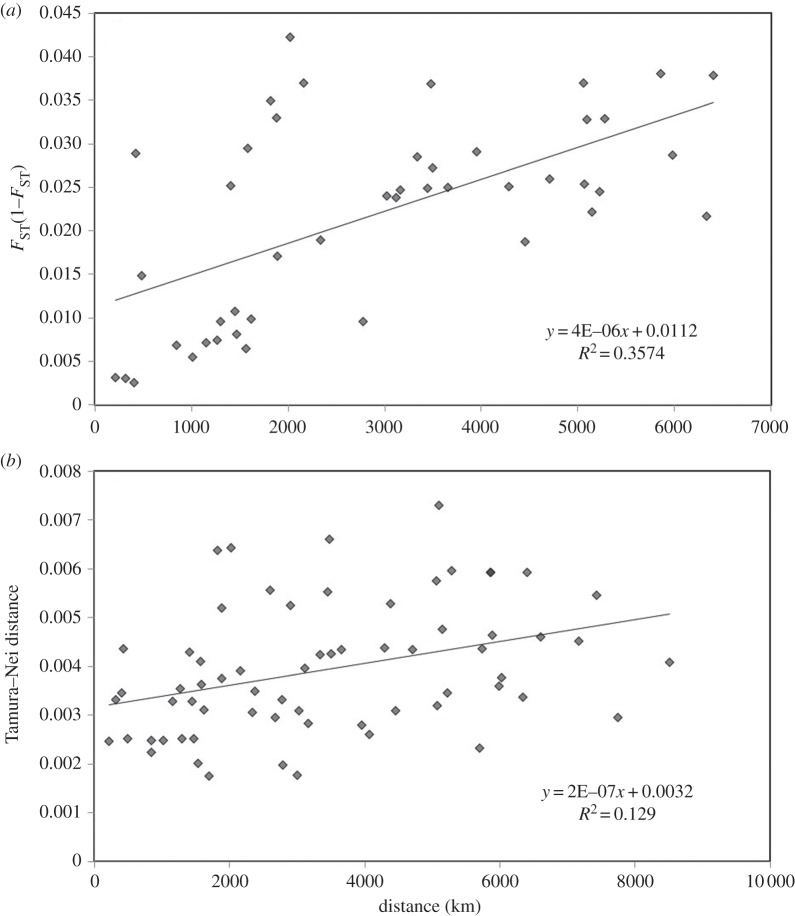
Associations between genetic and geographical distance based on (*a*) microsatellite data, and (*b*) mitochondrial DNA data.

The PCoA plot of pairwise genetic distance between sample collections clearly separated the Atlantic and the Mediterranean, but showed a greater level of division among the Mediterranean than the Atlantic sampling sites (figure 2*a*). There was a significant association between genetic and geographical distance across all samples (*R*^2^=0.357, *p*=0.002, figure 3*a*). This was also observed within the Atlantic (*R*^2^=0.333, *p*=0.047), but not within the Mediterranean (*R*^2^=0.087, *p*=0.383). Hierarchical AMOVA (table 3) showed significant variation between the Atlantic and the Mediterranean groups (2.09%), and significant variation among populations within these regions (1.78%), although within population genetic variation was greatest (96.13%).

**Table 3.** Hierarchical AMOVA of microsatellite (top) and mitochondrial data (bottom).

	source of	total	per cent			
data	variation	variation	of total	*F*_CT_	*F*_SC_	*F*_ST_
microsatellite	among seas	0.07716	2.09	0.02091 (*p*=0.00475)		
	among samples within seas	0.06568	1.78		0.01818 (*p*<0.0000)	
	within sample collections	3.54666	96.13			0.03872 (*p*<0.0000)
	total	3.68950				
mitochondrial DNA	among seas	0.11.329	12.56	0.12564 (*p*=0.01772)		
	among sample collections within seas	0.16439	18.23		0.20851 (*p*<0.0000)	
	within sample collections	0.62401	69.20			0.30796 (*p*<0.0000)
	total	0.90170

### 4.2 Microsatellite data: clustering analysis

Analysis of the complete microsatellite dataset in Structure initially divided the individuals into two clusters (electronic supplementary material, S3). The first cluster included the samples from the Atlantic, with all individuals demonstrating admixture coefficients showing more than 80% membership to this cluster, reflecting clear common ancestry. Similarly, individuals from Sardinia, Crete and the Adriatic also demonstrated admixture coefficients showing more than 80% membership to a second cluster, suggesting clear division between these two clusters. The Mallorca samples demonstrated some degree of admixture between these groups, exhibiting coefficients with an average 73% membership to the first Atlantic-dominated cluster and 27% membership to the second Mediterranean-dominated cluster. Subsequent hierarchical analysis of subsets of the data identified additional clusters corresponding to sampling locations within the Mediterranean, namely Crete, the Adriatic, Sardinia and a Mallorcan cluster that also incorporated the single individual from Tenerife. No evidence of genetic subdivision was found across the Atlantic samples.

### 4.3 Mitochondrial DNA data analysis: summary statistics and population differentiation

The alignment of 276 partial control region sequences comprised 412 bp, 26 haplotypes and 17 variable sites (accession numbers: KM873790–KM874065). Haplotype diversity ranged from 0.589 (Crete) to 0.815 (Western Channel), while rarefied haplotype richness ranged from 3.749 (Crete) to 8.322 (Portugal). Values of nucleotide diversity were low, ranging from 0.002 (Cyprus, Bristol, Scotland, Norway) to 0.004 (Portugal, Western Channel, Sardinia; table 4). Overall, significant genetic differentiation was observed (global *Φ*_ST_=0.308, *p*<0.001). Pairwise *Φ*_ST_ values ranged from −0.146 to 0.600, and were similar to the mt*F*_ST_ values (table 5). Most pairwise comparisons involving a Mediterranean population showed significant differentiation, and non-significant results involving Norway and Cyprus should be considered in the light of low sample size. No significant genetic structure was found among Atlantic collections.

**Table 4.** Mitochondrial DNA summary statistics. *n*, number of individuals; *H*_*n*_, number of haplotypes; *H*_*r*_, allelic richness; *h*, haplotype diversity; *π*, nucleotide diversity; s.d., standard deviation is in brackets; *D*, Tajima’s *D* value; *F*, Fu’s *F*_*s*_ value. *p<0.05, ^**^p<0.001, ^***^p<0.001.

sample collection	code	*n*	*H*_n_	*H*_r_	*h* (±s.d.)	*π*(±s.d.)	*D*	*F*
Norway	NOR	4	2	—	0.667(±0.204)	0.002(±0.002)	1.633	0.54
Scotland	SCO	23	5	4.913	0.676(±0.062)	0.002(±0.002)	−0.897	−1.096
North Sea	NSE	25	6	5.74	0.703(±0.071)	0.003(±0.002)	−0.92	−1.619
south Ireland	IRE	18	6	—	0.680(±0.109)	0.003(±0.002)	−0.336	−2.350*
Bristol Channel	BRI	27	5	4.626	0.678(±0.054)	0.002(±0.002)	−0.76	−0.893
Western Channel	WES	24	7	6.75	0.815(±0.045)	0.004(±0.003)	−0.31	−1.37
Portugal	POR	30	10	8.322	0.805(±0.050)	0.004(±0.003)	−1.157	−4.237^**^
Mallorca	MAL	40	8	5.97	0.659(±0.070)	0.003(±0.002)	−0.528	−2.234
Sardinia	SAR	22	5	5	0.732(±0.068)	0.004(±0.003)	1.247	0.2
Adriatic	ADR	27	5	4.63	0.715(±0.047)	0.003(±0.002)	−0.563	−0.653
Crete	CRE	29	4	3.749	0.589(±0.075)	0.003(±0.002)	0.78	0.596
Cyprus	CYP	6	3	—	0.600(±0.215)	0.002(±0.002)	−1.233	−0.189
all		276	26	—	0.817(±0.015)	0.004(±0.003)	−1.032	−16.573^***^

**Table 5.** Mitochondrial pairwise differentiation among 12 sampling localities. Below diagonal, pairwise *Φ*_ST_ values. Above diagonal, pairwise mt*F*_ST_ values. Values in bold were significant at the 95% CI, and those marked with asterisks (***) remained significant after sequential Bonferroni corrections.

locality	Norway	Scotland	North Sea	south Ireland	Bristol Channel	Western Channel	Portugal	Mallorca	Sardinia	Adriatic	Crete	Cyprus
Norway	—	−0.145	−0.110	−0.056	−0.136	0.017	−0.094	−0.059	0.110	0.143	**0**.**390**	**0**.**314**
Scotland	−0.146	—	−0.014	0.029	−0.035	0.047	−0.014	0.021	**0**.**152**	**0**.186^***^	**0**.370^***^	**0**.**306**
North Sea	−0.117	0.007	—	−0.027	−0.013	0.028	−0.001	**0**.**089**	**0**.**155**	**0**.137^***^	**0**.356^***^	**0**.**276**
south Ireland	−0.090	0.028	−0.039	—	0.021	0.029	0.034	**0**.**160**	**0**.180^***^	**0**.**121**	**0**.371^***^	**0**.**284**
Bristol Channel	−0.143	−0.028	0.007	0.022	—	0.044	−0.004	0.035	**0**.**153**	**0**.166^***^	**0**.367^***^	**0**.**302**
Western Channel	−0.054	0.019	0.034	0.033	0.030	—	0.030	**0**.**134**	**0**.149^***^	**0**.143^***^	**0**.302^***^	**0**.**228**
Portugal	−0.130	−0.022	0.000	0.012	−0.006	0.017	—	0.031	**0**.**116**	**0**.145^***^	**0**.303^***^	**0**.**232**
Mallorca	−0.110	0.003	0.036	0.054	0.014	**0**.**067**	0.011	—	**0**.**123**	**0**.242^***^	**0**.374^***^	**0**.338^***^
Sardinia	0.180	**0**.297^***^	**0**.246^***^	**0**.240^***^	**0**.280^***^	**0**.275^***^	**0**.249^***^	**0**.**187**	—	**0**.200^***^	**0**.344^***^	**0**.295^***^
Adriatic	**0**.**239**	**0**.326^***^	**0**.2226^***^	**0**.209^***^	**0**.301^***^	**0**.304^***^	**0**.254^***^	**0**.252^***^	**0**.**150**	—	**0**.270^***^	**0**.**177**
Crete	**0**.581^***^	**0**.597^***^	**0**.566^***^	**0**.568^***^	**0**.600^***^	**0**.555^***^	**0**.516^***^	**0**.549^***^	**0**.511^***^	**0**.412^***^	—	−0.054
Cyprus	**0**.**486**	**0**.491^***^	**0**.424^***^	**0**.428^***^	**0**.495^***^	**0**.400^***^	**0**.352^***^	**0**.425^***^	**0**.392^***^	**0**.**283**	0.009	—

The PCoA plot of pairwise genetic distance showed the close clustering of the Atlantic sample collections, whereas the Mediterranean groups appear to be relatively distinct. The Mallorca collection remained an exception, as it clustered more closely with the Atlantic (figure 2*b*). There was a significant association between genetic and geographical distance across all sample collections (*R*^2^=0.129, *p*=0.016; figure 3*b*), but not within the Atlantic (*R*^2^=0.039, *p*=0.348), or within the Mediterranean (*R*^2^=0.149, *p*=0.167). Hierarchical AMOVA (table 3) showed significant variation between the Atlantic and the Mediterranean groups (12.56%), and significant variation among populations within these regions (18.23%), although within population genetic variation was greatest (69.20%).

### 4.4 Mitochondrial DNA data analysis: haplotype network and demographic analysis

**Figure 4.**
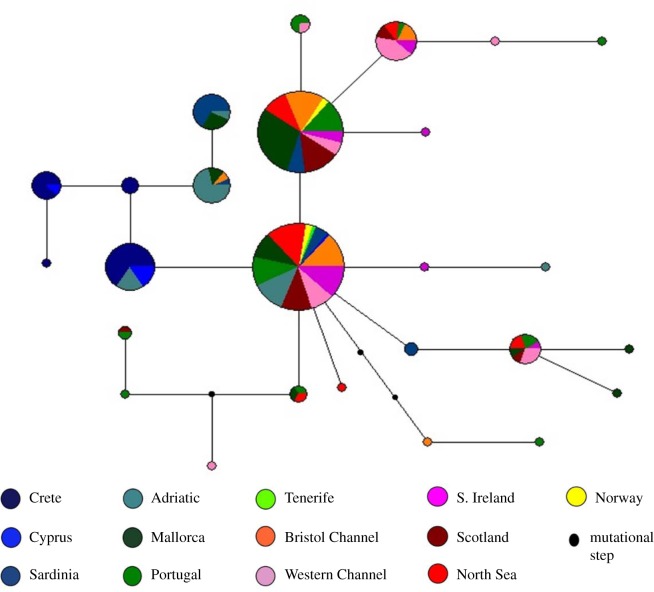
Haplotype network of mitochondrial control region sequences. The sizes of the circles in the network are proportional to the frequency of the haplotype in the dataset, and are coloured according to the sample collections. Ambiguous links were broken according to phylogenetic relationships estimated from the consensus maximum-likelihood tree.

The haplotype network demonstrated that two common haplotypes predominate in the Atlantic, but are also present in the Mediterranean (figure 4). There was little evidence of population structure in the Northeast Atlantic, as haplotypes did not appear to assort by sample location. However, a number of closely related haplotypes were unique to the eastern Mediterranean, supporting the distinctiveness of the sample collections from this region, particularly those from the eastern basin (Crete and Cyprus).

Demographic analyses showed that the Atlantic sample collections had unimodal distributions with negative Tajima’s *D* and Fu’s *F* values, although only *F* values related to southern Ireland and Portugal were significant, providing evidence of a population expansion (table 2; electronic supplementary material, S4). Sample collections from Sardinia and Crete had positive *D* and *F* values and bimodal distributions, indicating demographic stability. The remaining sample collections generally yielded non-significant results. The BSP analysis (electronic supplementary material, S5) indicates slowly declining population sizes within the Mediterranean, with evidence of population growth in the past 100 000–500 000 years (with the exception of the Sardinian sample that shows a recent decline in effective population size). This contrasts with the Northeast Atlantic, in which the samples generally demonstrate a more sustained period of much greater population increase, dating back over 500 000–1 250 000 years.

### 4.5 Sex-biased dispersal

The mt*F*_ST_ was significantly greater than *F*_ST_ across the sampling area (paired *t*-test; *t*=6.487, *p*<0.001; tables 2 and 5) consistent with male dispersal and female philopatry. In direct comparisons of male and female microsatellite data, males showed a higher *F*_IS_, lower *F*_ST_, lower relatedness values, lower mAIc and higher vAIC, all consistent with male-biased dispersal. However, only *F*_ST_ and mAIc showed a significant difference between the sexes (table 6).

**Table 6.** Results of the five tests of sex bias dispersal using microsatellite markers.

sex	*n*	*F*_IS_	*F*_ST_	relatedness	mAIc	vAIc
male	186	0.0203	0.0202	0.0388	−0.91583	11.69206
female	253	−0.0156	0.0321	0.0631	0.67330	10.66518
*p*-value		0.0232	0.1610	0.1019	0.0001	0.0764

## 5. Discussion

This study represents the most detailed analysis of genetic variation from a species in the largest family of sharks, the Scyliorhinidae, incorporating samples from a wide trans-European and Mediterranean area. The results showed high gene flow in the Northeast Atlantic Ocean, a region of connectivity between the Atlantic and the Mediterranean populations in the western part of the Mediterranean Sea, and genetic differences among populations across the Mediterranean basin. Indications of male-biased dispersal and female philopatry were also identified, supporting growing evidence of sex-based differences in dispersal and behaviour in elasmobranchs [76,77].

A lack of temporal variation in the Western English Channel over an 8-year sampling period supported the stability in population structure in this region over time, which remains a key factor in interpreting genetic data. By contrast, the null hypothesis of panmixia in *S. canicula* was rejected by analyses for both mtDNA control region sequence and nuclear microsatellite markers when considering all the sample collections across the Northeast Atlantic and the Mediterranean. This indicates that traits associated with limited dispersal potential may have played an important role in limiting gene flow, a finding that is becoming increasingly common in coastal and demersal sharks [13,30]. A significant pattern of isolation by distance (IBD) was also identified by both sets of markers, primarily related to significant genetic differences between the Atlantic and the Mediterranean, especially eastern Mediterranean, populations. Such regional variation could also be linked to ontogenetic differences of *S*. *canicula*, with length and age at sexual maturity attained earlier in the Mediterranean than in the Atlantic [20,25,33], so that genetic and morphological differences appear to coincide.

The mtDNA data identified two main groups of haplotypes; the first included the highest frequency haplotypes, central to the network that were predominantly associated with samples from the Northeast Atlantic and the Balearic Islands. The second includes haplotypes unique to specimens found in central and eastern parts of the Mediterranean (figure 4). Furthermore, pairwise *Φ*_ST_ revealed highly significant differences between biogeographically distinct regions of the Mediterranean: the western Mediterranean, eastern Mediterranean and Adriatic Sea. Such a trend runs counter to previous studies on elasmobranchs in these waters, where a lack of divergence has been found [44,78,79]. However, similar patterns of population subdivision have been described by the only other investigation of *S. canicula* [23], where variation within the cytochrome oxidase *I* gene was analysed from predominantly Mediterranean individuals. That study did not find evidence of population structure at such a fine geographical scale, perhaps due to the relatively conservative nature of the protein coding gene region used [80]. Additionally, Barbieri *et al*. [23] grouped distant areas of the Mediterranean owing to sample size restrictions, potentially reducing the resolution of their analysis. Application of microsatellite loci in the current study also supported evidence of genetic sub-division between *S. canicula* in the eastern and western Mediterranean. Indeed, the results suggest differentiation at an even smaller scale, between sample collections separated by less than 500 km in the western Mediterranean (Balearic Islands and Sardinia).

The pattern of highly divided population structure across the Mediterranean contrasts very sharply with results from the Northeast Atlantic. Regardless of the markers used, or the analytical approach, there was no evidence of significant genetic differences between sample collections originating from this region. Individuals from Norway and Africa demonstrated microsatellite alleles and haplotypes that were generally common across the Atlantic, suggesting little evidence of population structure, even at the most extreme latitudinal ranges considered. This lack of genetic differences across the Atlantic waters does not conform to expectations from typical life-history characteristics of small, demersal elasmobranchs that suggest low dispersal potential [81]. Nevertheless, similar patterns of little genetic evidence of population structure have also been reported for the starry ray (*Amblyraja radiata*) in the north Atlantic over comparable scales [81,82]. Perhaps both of these species correspond to a more typical pattern of marine species population structure with large effective population sizes and high gene flow within the Atlantic that minimize the effects of genetic drift and lead to low levels of population structure that are difficult to detect [83,84].

While nuclear and mtDNA data generally exhibited highly concordant results, differing signals of population structure were identified around Mallorca, with mtDNA suggesting a close similarity with the Atlantic group (figure 2*b*), and microsatellite markers indicating a more intermediate position between the Atlantic and the Mediterranean groups (figure 2*a*). This result is consistent with the Balearic Islands representing an important region of secondary contact. There is strong evidence that many marine species have invaded the western Mediterranean through the Strait of Gibraltar since the last glacial maximum, bringing previously allopatric lineages into contact [47,85–87]. The differences in the modes of inheritance between the two marker types may also explain the differing patterns of population structure identified, with the smaller effective population size of mtDNA potentially leading to more rapid genetic drift and the fixation of Atlantic haplotypes within the populations of the western Mediterranean.

### 5.1 Phylogeography

There have been long-standing hypotheses suggesting that the Strait of Gibraltar (the so-called ‘Pillars of Hercules’) or the Almeria–Oran Front represents phylogeographic barriers that shape the biogeographical patterns of marine Atlantic–Mediterranean organisms (reviewed in [47]). A number of molecular studies corroborate these scenarios with genetic discontinuity between the Atlantic and the Mediterranean populations occurring at the Strait of Gibraltar [88–92]. However, since the opening of the Strait of Gibraltar occurred at the end of the Messinian salinity crisis, its status as a current barrier to gene flow has been questioned. This study does not support a genetic discontinuity across the Strait, as an important zone of secondary contact between these populations is actually present in the Balearic Sea (followed by increasing genetic distinctiveness in the more isolated and semi-enclosed regions of the eastern Mediterranean, perhaps corresponding to relictual populations). Similar zones of secondary contact between the Mediterranean and the Northeast Atlantic stocks are also observed in other fish, such as the Atlantic bonito and the swordfish [93,94], suggesting this may be a common phylogeographic scenario.

The Mediterranean and the Northeast Atlantic share a history of inter-connectedness and have a large number of species in common. However, some evidence of contrasting demographic signatures was detected between the Atlantic and the Mediterranean populations of *S. canicula*. The Atlantic populations typically showed patterns of population expansion, while the Mediterranean appeared much more stable. This could be explained by the long-term stability and the consistent existence of suitable habitat in the Mediterranean during the Pleistocene glacial and interglacial cycles, as has been observed for other species [95,96]. Many of the Mediterranean basins are relatively deep, potentially providing suitable habitat for *S. canicula* during climatically driven sea-level fluctuations, allowing its persistence [97]. Recolonization of North Atlantic shelf habitats may have been rapid over the current interglacial cycle, meaning that it would not have promoted genetic discontinuity among regions. Interestingly, no evidence of northern refugia was supported by our data, in contrast to other marine taxa [43–45,98,99]. This lack of spatial association is also shown in the haplotype distribution where the two main haplotypes are shared by individuals caught along the Atlantic coast (figure 4). Together these data are suggestive of a relatively sudden population expansion. The apparent absence of a northward decrease in genetic diversity (table 1) is, however, not generally supportive of the ‘leading edge hypothesis’ [100], where latitudinal genetic variation is reduced in recently colonized populations owing to stochastic processes.

### 5.2 Sex-biased dispersal

Despite obvious congruence in patterns of population structure identified between the mtDNA and microsatellite data (tables 2 and 5), global testing across all populations demonstrated significant differences that were consistent with male-biased dispersal and female philopatry in *S. canicula*. This supports growing evidence of sex-based differences in dispersal and behaviour in elasmobranchs more widely [76,77]. However, there are limitations with the tests employed in this study. In the comparison of mtDNA and nuclear DNA markers, the reduced effective population size of mtDNA leads to expectations of greater *F*_ST_ values, regardless of differences in sex-biased dispersal. Therefore, the recent approach of Daly-Engel *et al*. [35] was used; they suggested this comparison is robust with the use of numerous polymorphic nuclear markers that provide strong statistic power to detect population structure [101]. If population sizes are stable, it will also decrease the chance that differences in mtDNA and nuclear DNA are driven purely by variation in marker effective population size [35].

In order to overcome the limitations with comparisons of mtDNA and nuclear DNA, an additional suite of tests for sex-biased dispersal that focus on the microsatellite markers alone was conducted. These tests have a number of assumptions, including non-overlapping generations, equal sample size, juvenile dispersal and equal sex ratios [75] that are not necessarily satisfied in this case. However, the methods do provide a valuable way of interrogating the data and have been used in similar studies [102–104].

The apparent male-biased dispersal supports a long history of work demonstrating strong sex-based differences in the behaviour of the small-spotted catshark. It was the first elasmobranch species for which systematic analyses of unequal sex ratios in trawl catches provided clear evidence for unisexual aggregations [105,106], with recent work suggesting that this is the result of sexual harassment by males [107,108]. Finally, our results also support the commonly made association between the reproductive strategies of sharks and marine mammals that much greater investment in reproduction by females than males is at the evolutionary root of these differences in behaviour [27,35].

### 5.3 Conservation implications

The assessment and management of shark stocks is not well established, in part, because many characteristics such as dispersal or migratory behaviour are not fully understood [37]. Additional factors such as female philopatry, sexual segregation and sex-biased dispersal should be better considered in any management regimes, as spatially focused fisheries could result in the differential exploitation of sexes. The results of this study clearly show the potential for *S. canicula* to form multiple stocks within its distributional range. This has important implications for sustainable management, as effective conservation measures may need to be implemented at the level of the demographic unit to ensure long-term stock viability in the face of exploitation [109]. This is especially relevant in the case of the northern Adriatic Sea stock, which appears to have undergone dramatic declines in abundance [26], but for which recovery may not be as simple as immigration from proximate regions.

## Supplementary Material

SM1 Sample Details

## Supplementary Material

SM2 Microsatellite Summary Statistics

## Supplementary Material

SM3 Heierarchical STRUCTURE analysis

## Supplementary Material

SM4 Mis-match Analysis

## Supplementary Material

SM5 Bayesian Skyline Plots

## Data accessibility

DNA sequences generated in this study have Genbank accessions: KM873790–KM874065.
