# Supplementary material for: A tale of two seas: contrasting patterns of population structure in the small-spotted catshark across Europe
Source: R Soc Open Sci. 2014 Nov 12;1(3):140175. doi: 10.1098/rsos.140175 (PMC4448844; doi:10.1098/rsos.140175)
Supplement: SM2 Microsatellite Summary Statistics [file rsos140175supp2.doc]

**Supplementary Material 2. Microsatellite summary statistics.** Number of alleles, Number of observations (N), observed (H_O_) and expected (H_E_) heterozygosities, allelic richness (R_S_), probability of conformance to Hardy-Weinberg equilibrium (HWE) and Weir & Cockerham’s inbreeding coefficient (F_IS_). Significant deviation from conformance to HWE after sequential Bonferroni correction (initial correction level was made across loci within a population; 0.05/12) is indicated in bold. The single sample from Africa/the Canaries is excluded from this table due to the small sample size. Allelic richness values were calculated after excluding the Norwegian sample due to the small sample size, so rarefaction standardised to the level of North Sea collection (N = 25). Per locus global F_ST_ estimates (F_ST_) are also included at the bottom of the table.

| **Sample Collection** |  | **Scan15** | **Scan13** | **Scan04** | **Scan10** | **Scan14** | **Scan16** | **Scan03** | **Scan09** | **Scan02** | **Scan06** | **Scan05** | **Scan12** | **Mean** |
| --- | --- | --- | --- | --- | --- | --- | --- | --- | --- | --- | --- | --- | --- | --- |
| CRETE | No. alleles  N  H_O_  H_E_  R_S_  HWE  F_IS_ | 5  35  0.543  0.5  4.63  0.5917  -0.0866 | 5  35  0.4  0.375  4.87  0.6545  -0.0685 | 3  35  0.4  0.409  2.69  0.3969  0.0216 | 5  34  0.676  0.641  4.89  0.5934  -0.0556 | 9  33  0.636  0.73  8.28  0.6063  0.1301 | 8  35  0.771  0.721  7.18  0.6039  -0.0712 | 12  34  0.647  0.749  10.82  0.6027  0.1383 | 4  35  0.343  0.465  3.37  0.1169  0.2662 | 6  35  0.6  0.704  5.59  0.2063  0.1495 | 7  35  0.571  0.605  6.36  0.6966  0.0569 | 3  35  0.514  0.457  2.69  0.2164  -0.1281 | 4  35  0.771  0.631  4.00  0.2752  -0.2265 | 5.92  34.668  0.5823  0.5727  5.45  0.6016 |
| ADRIATIC | No. alleles  N  H_O_  H_E_  R_S_  HWE  F_IS_ | 6  46  0.717  0.713  5.46  0.0547  -0.0061 | 7  47  0.447  0.443  5.41  0.8102  -0.0094 | 3  46  0.565  0.451  2.52  0.1608  -0.2560 | 5  47  0.766  0.668  4.65  0.1721  -0.1476 | 8  45  0.667  0.760  6.83  0.2765  0.1238 | 7  45  0.689  0.732  6.64  0.5951  0.0596 | 9  47  0.787  0.853  8.48  0.5590  0.0783 | 3  47  0.277  0.244  2.51  1.0000  -0.1369 | 8  44  0.727  0.798  7.34  0.0227  0.0893 | 8  45  0.778  0.827  7.02  0.0808  0.0607 | 4  47  0.511  0.460  3.46  0.2154  -0.1123 | 5  46  0.717  0.589  4.30  0.5573  -0.2212 | 6.08  45.999  0.6373  0.6282  5.39  0.0681 |
| SARDINIA | No. alleles  N  H_O_  H_E_  R_S_  HWE  F_IS_ | 6  58  0.672  0.622  5.65  0.7686  -0.0823 | 8  59  0.271  0.280  5.82  0.3545  0.0323 | 3  59  0.339  0.419  2.41  0.2828  0.1914 | 6  59  0.542  0.481  4.80  0.9769  -0.1283 | 10  56  0.696  0.806  8.42  0.1995  0.1368 | 7  58  0.741  0.792  6.76  0.2119  0.0645 | 8  58  0.707  0.786  7.20  0.3451  0.1017 | 2  59  0.186  0.171  2.00  1.0000  -0.0943 | 8  59  0.780  0.777  7.23  0.5697  -0.0040 | 9  59  0.814  0.805  7.63  0.6188  -0.0102 | 4  59  0.661  0.646  3.65  0.7325  -0.0235 | 5  59  0.542  0.516  3.81  0.3992  -0.0522 | 6.33  58.499  0.5793  0.5917  5.45  0.7946 |
| MALLORCA | No. alleles  N  H_O_  H_E_  R_S_  HWE  F_IS_ | 5  51  0.588  0.551  4.92  0.8748  -0.0691 | 4  52  0.231  0.229  3.38  0.5748  -0.0074 | 4  52  0.654  0.535  3.31  0.1261  -0.2241 | 7  51  0.431  0.486  4.87  0.1538  0.1140 | 8  51  0.667  0.738  6.88  0.4287  0.0974 | 8  51  0.882  0.834  7.41  0.8870  -0.0586 | 12  50  0.680  0.731  8.91  0.7044  0.0706 | 2  52  0.135  0.160  2.00  0.3144  0.1580 | 7  50  0.840  0.764  6.39  0.5178  -0.1008 | 9  52  0.712  0.767  7.92  0.6533  0.0734 | 4  51  0.412  0.355  3.89  0.9213  -0.1615 | 4  52  0.615  0.594  3.95  0.4992  -0.0359 | 6.17  51.251  0.5706  0.5620  5.32  0.8064 |
| PORTUGAL | No. alleles  N  H_O_  H_E_  R_S_  HWE  F_IS_ | 7  29  0.828  0.789  6.96  0.7274  -0.0492 | 7  29  0.552  0.531  6.43  0.8720  -0.0394 | 4  29  0.483  0.586  3.86  0.1451  0.1791 | 5  29  0.448  0.575  4.84  0.2040  0.2239 | 10  29  0.759  0.851  9.69  0.0633  0.1098 | 7  29  0.759  0.768  6.84  0.3878  0.0120 | 7  29  0.621  0.729  6.98  0.2369  0.1508 | 2  29  0.379  0.313  2.00  0.5496  -0.2174 | 8  29  0.862  0.789  7.71  0.0776  -0.0938 | 8  29  0.759  0.787  7.71  0.1080  0.0367 | 5  29  0.483  0.479  4.71  0.6841  -0.0077 | 7  29  0.690  0.728  6.69  0.0922  0.0541 | 6.42  29  0.6353  0.6605  6.20  0.0756 |
| WESTERN CHANNEL | No. alleles  N  H_O_  H_E_  R_S_  HWE  F_IS_ | 10  105  0.733  0.774  7.09  0.6621  0.0533 | 8  109  0.596  0.535  5.52  0.5387  -0.1149 | 4  109  0.523  0.505  3.12  0.9084  -0.0358 | 6  107  0.411  0.497  4.31  0.0198  0.1730 | 13  106  0.811  0.830  9.04  0.4816  0.0230 | 7  106  0.840  0.838  6.93  0.1525  -0.0024 | 11  103  0.563  0.606  7.66  0.1122  0.0718 | 3  110  0.191  0.189  2.22  1.0000  -0.0102 | 11  92  0.870  0.811  8.47  0.6032  -0.0725 | 9  105  0.819  0.809  7.30  0.7471  -0.0130 | 4  108  0.602  0.517  3.67  0.4403  -0.1644 | 6  109  0.697  0.695  5.26  0.0294  -0.0039 | 7.67  105.754  0.6380  0.6339  5.88  0.1851 |
| BRISTOL CHANNEL | No. alleles  N  H_O_  H_E_  R_S_  HWE  F_IS_ | 7  46  0.717  0.767  6.54  0.6894  0.0654 | 5  45  0.556  0.500  4.07  0.4858  -0.1117 | 3  46  0.500  0.506  2.95  0.0972  0.0124 | 5  45  0.556  0.551  4.06  0.2173  -0.0087 | 10  45  0.800  0.857  8.95  0.0429  0.0674 | 6  44  0.795  0.806  5.99  0.0774  0.0138 | 8  46  0.630  0.621  7.26  0.9094  -0.0160 | 3  46  0.152  0.145  2.75  1.0000  -0.0535 | 9  46  0.870  0.841  8.41  0.3464  -0.0339 | 9  43  0.791  0.789  7.99  0.6898  -0.0018 | 4  45  0.600  0.554  3.53  0.5251  -0.0839 | 5  43  0.721  0.635  4.37  0.5343  -0.1366 | 6.17  45.002  0.6407  0.6311  5.57  0.3087 |
| IRELAND | No. alleles  N  H_O_  H_E_  R_S_  HWE  F_IS_ | 9  50  0.840  0.773  7.14  0.4254  -0.0877 | 6  50  0.480  0.492  4.44  0.1453  0.0241 | 4  50  0.540  0.517  3.72  0.4556  -0.0442 | 4  49  0.551  0.554  3.87  0.7359  0.0054 | 12  50  0.840  0.860  10.09  0.8722  0.0233 | 7  49  0.776  0.832  6.74  0.2880  0.0689 | 9  50  0.520  0.618  7.66  0.0222  0.1594 | 2  50  0.120  0.114  1.98  1.0000  -0.0538 | 10  50  0.900  0.815  8.83  0.1221  -0.1061 | 10  50  0.800  0.852  8.91  0.5384  0.0613 | 4  50  0.460  0.489  3.72  0.3775  0.0608 | 6  49  0.571  0.652  4.92  0.0060  0.1250 | 6.92  49.750  0.6165  0.6306  6.00  0.0581 |
| SCOTLAND | No. alleles  N  H_O_  H_E_  R_S_  HWE  F_IS_ | 9  50  0.740  0.778  7.30  0.6048  0.0490 | 7  50  0.580  0.516  5.52  0.8838  -0.1264 | 3  50  0.340  0.346  2.86  0.8237  0.0177 | 5  48  0.563  0.480  4.63  0.3762  -0.1745 | 10  50  0.800  0.855  8.66  0.0759  0.0651 | 8  49  0.735  0.823  7.23  0.1284  0.1079 | 10  49  0.612  0.623  7.95  0.7205  0.0167 | 3  50  0.060  0.059  2.21  1.0000  -0.0138 | 10  50  0.860  0.827  8.90  0.1223  -0.0400 | 10  50  0.920  0.809  8.73  0.7094  -0.1384 | 5  50  0.440  0.540  4.18  0.2412  0.1867 | 5  50  0.580  0.665  4.46  0.7074  0.1293 | 7.08  49.665  0.6025  0.6100  6.02  0.5828 |
| NORTH SEA | No. alleles  N  H_O_  H_E_  R_S_  HWE  F_IS_ | 8  25  0.840  0.779  7.88  0.6774  -0.0804 | 3  25  0.320  0.318  3.00  0.5825  -0.0079 | 2  25  0.440  0.507  2.00  0.6877  0.1344 | 5  25  0.600  0.538  4.96  0.4873  -0.1180 | 8  25  0.960  0.854  7.96  0.1221  -0.1272 | 6  24  0.792  0.848  6.00  0.9277  0.0672 | 7  25  0.680  0.736  6.92  0.6842  0.0780 | 2  25  0.160  0.150  2.00  1.0000  -0.0667 | 8  24  0.917  0.801  8.00  0.4983  -0.1474 | 9  24  0.833  0.829  9.00  0.9926  -0.0055 | 3  25  0.520  0.448  3.00  0.8428  -0.1642 | 6  25  0.840  0.692  5.92  0.5897  -0.2189 | 5.58  24.750  0.6585  0.6250  5.55  0.9803 |
| NORWAY | No. alleles  N  H_O_  H_E_  HWE  F_IS_ | 4  4  1  0.75  1.0000  -0.4118 | 4  4  0.5  0.75  0.3143  0.3684 | 2  4  0.25  0.25  -  - | 3  4  0.5  0.464  1.0000  -0.0909 | 3  4  0.5  0.679  1.0000  0.2941 | 5  4  0.75  0.857  0.6560  0.1429 | 4  4  0.5  0.786  0.3143  0.4000 | 1  4  0  0  -  - | 4  4  0.75  0.75  1.0000  0.0000 | 4  4  1  0.821  1.0000  -0.2632 | 2  4  0.75  0.536  1.0000  -0.5000 | 4  4  0.75  0.75  1.0000  0.0000 | 3.33  4  0.6042  0.6161  0.9994 |
| Total | No. alleles  N  H_O_  H_E_  Mean R_S_  HWE  F_IS_  F_ST_ | 11  497  0.720  0.738  6.79  0.3805  0.0281  0.0443 | 10  503  0.459  0.447  5.48  0.7314  -0.0197  0.0316 | 4  503  0.481  0.483  3.04  0.2576  0.0074  0.0234 | 8  496  0.534  0.544  5.08  0.0416  0.0121  0.0187 | 16  492  0.760  0.836  9.00  0.0000  0.0939  0.0312 | 9  492  0.785  0.829  6.89  0.0313  0.0497  0.0295 | 18  493  0.635  0.711  8.47  0.0144  0.1091  0.0262 | 6  505  0.186  0.194  2.32  0.2505  0.0335  0.0449 | 12  481  0.825  0.815  8.34  0.2789  -0.0125  0.0287 | 12  494  0.791  0.811  7.95  0.1343  0.0284  0.0230 | 5  501  0.535  0.514  3.72  0.5796  -0.0439  0.0208 | 8  499  0.663  0.664  4.95  0.5337  0.0026  0.0413 |  |
