# Supplementary material for: A tale of two seas: contrasting patterns of population structure in the small-spotted catshark across Europe
Source: R Soc Open Sci. 2014 Nov 12;1(3):140175. doi: 10.1098/rsos.140175 (PMC4448844; doi:10.1098/rsos.140175)
Supplement: SM3 Heierarchical STRUCTURE analysis [file rsos140175supp3.doc]

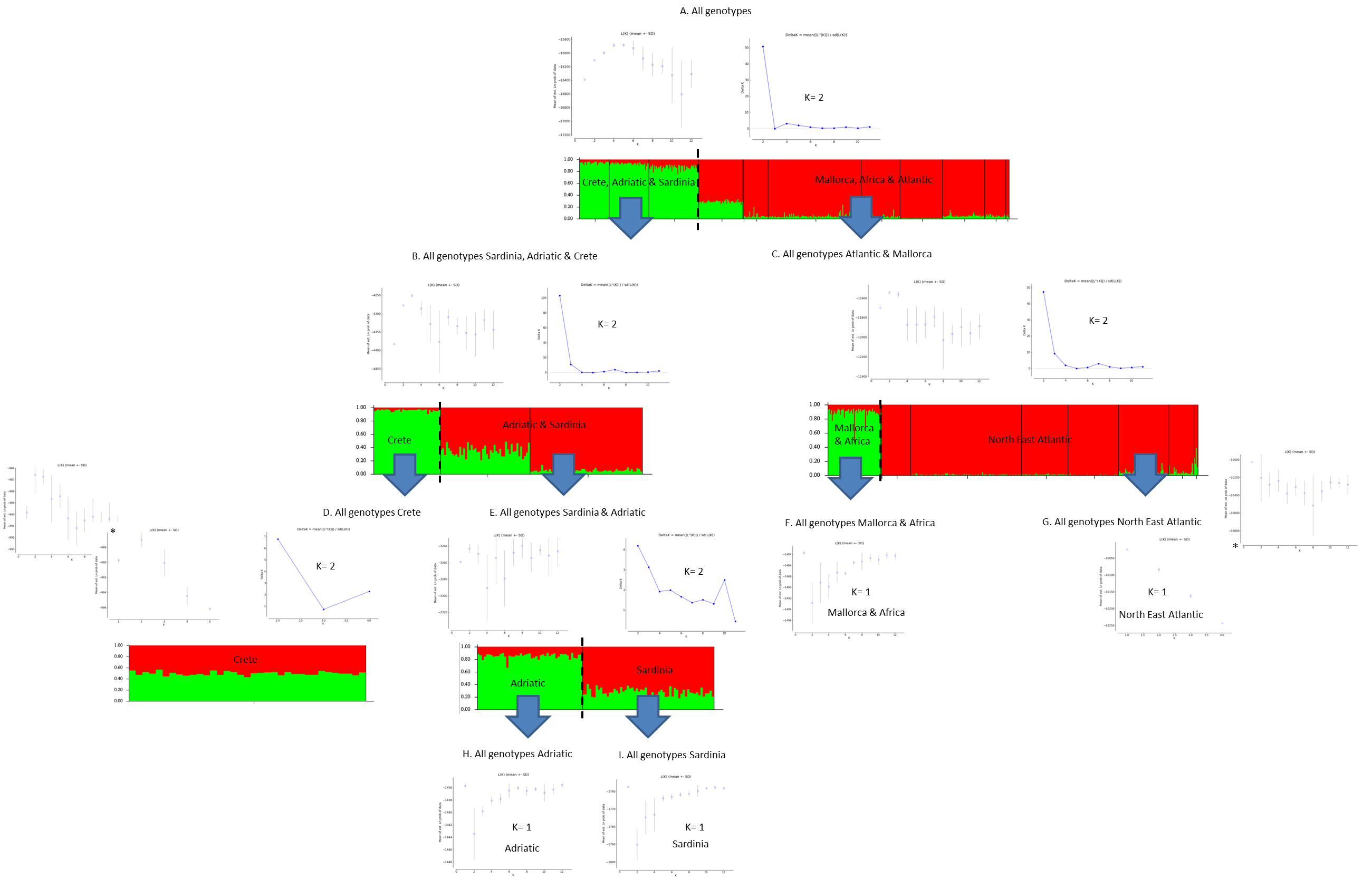
Supplementary Material 5. Hierarchical analysis in STRUCTURE. Each individual is represented by a thin horizontal line which is partitioned into *K* coloured segments, representing an individual’s estimated membership fractions in *K* clusters. The dashed lines represent how the data set was partitioned in each subsequent hierarchy of the analysis. Plots of absolute values of ln Pr(*X | K*) and *ΔK* are also shown. To judge the correct *K*, the *ΔK* method of Evanno *et al.* (2005) was applied.

* After the initial run with 1 million MCMC, if the estimation of likelihoods was highly variable between iterations, run lengths were increased to >5 million MCMC (10% burn-in), although the maximum number of clusters (K) was reduced to 5 in order to reduce computation time.
