# Supplementary material for: A tale of two seas: contrasting patterns of population structure in the small-spotted catshark across Europe
Source: R Soc Open Sci. 2014 Nov 12;1(3):140175. doi: 10.1098/rsos.140175 (PMC4448844; doi:10.1098/rsos.140175)
Supplement: SM4 Mis-match Analysis [file rsos140175supp4.ppt]

## Slide 1
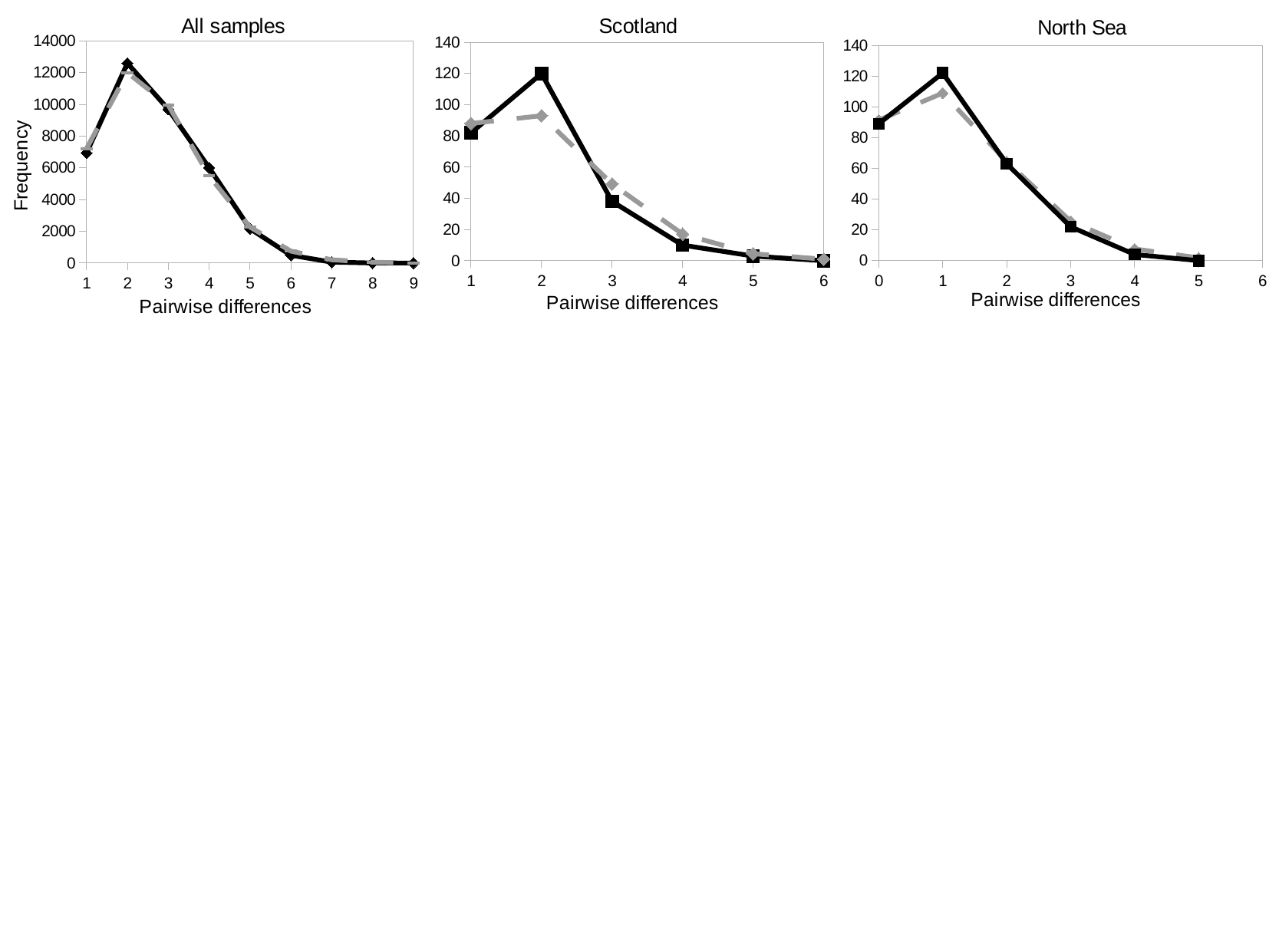

### Chart: Scotland
| Category | Observed | Simulated |
|---|---|---|
| 1 | 82.0 | 88.0 |
| 2 | 120.0 | 93.0 |
| 3 | 38.0 | 49.2 |
| 4 | 10.0 | 17.1 |
| 5 | 3.0 | 4.5 |
| 6 | 0.0 | 1.0 |
### Chart: North Sea
| Category | Column B | Column C |
|---|---|---|
### Chart: All samples
| Category | Observed | Simulated |
|---|---|---|
| 1 | 6939.0 | 7218.6 |
| 2 | 12599.0 | 12004.3 |
| 3 | 9693.0 | 9953.6 |
| 4 | 6006.0 | 5490.1 |
| 5 | 2167.0 | 2269.2 |
| 6 | 489.0 | 748.8 |
| 7 | 55.0 | 205.2 |
| 8 | 2.0 | 48.2 |
| 9 | 0.0 | 9.9 |

## Slide 2
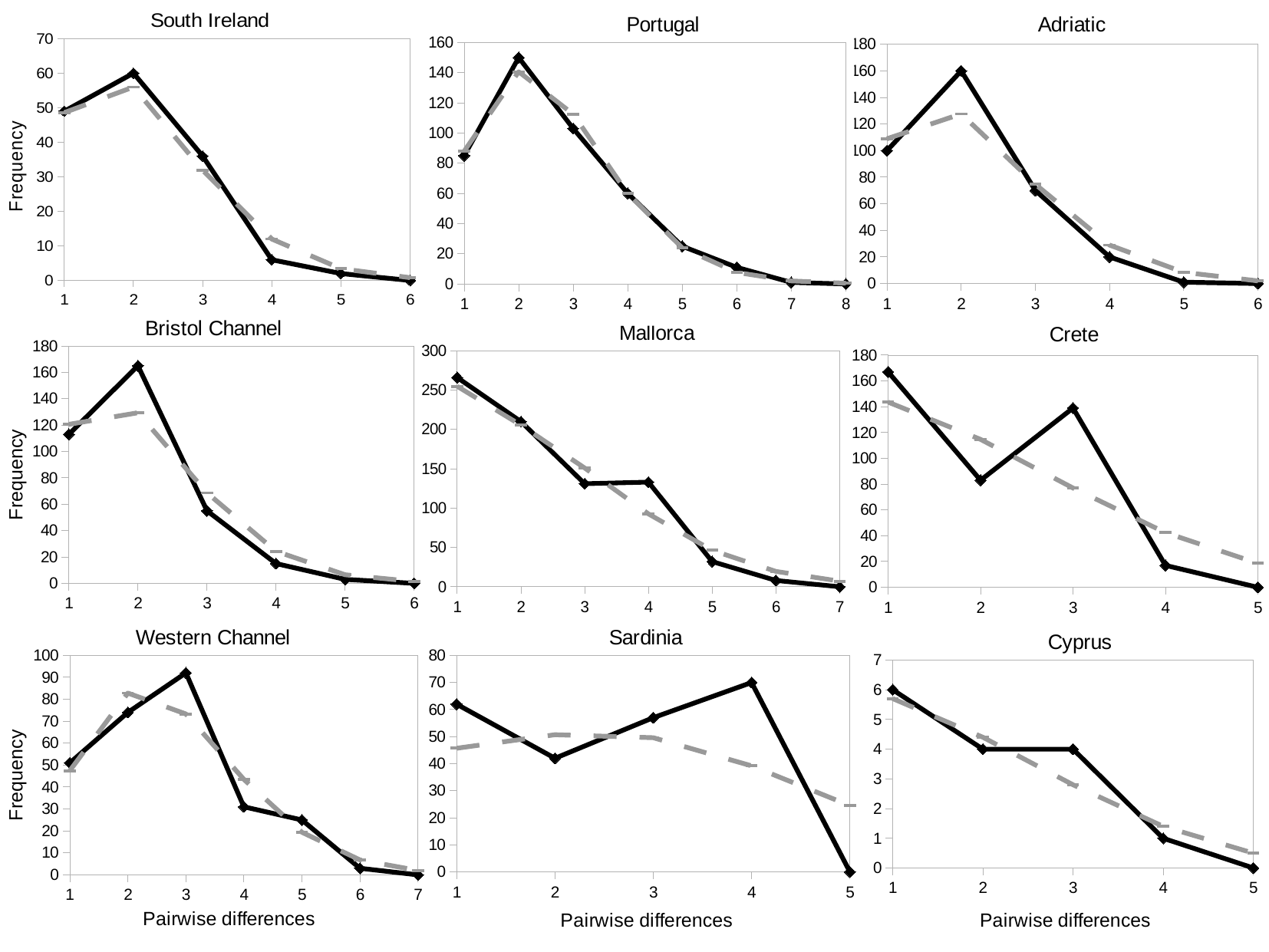

### Chart: South Ireland
| Category | Observed | Simulated |
|---|---|---|
| 1 | 49.0 | 48.6 |
| 2 | 60.0 | 56.0 |
| 3 | 36.0 | 31.9 |
| 4 | 6.0 | 12.0 |
| 5 | 2.0 | 3.4 |
| 6 | 0.0 | 0.8 |
### Chart: Adriatic
| Category | Observed | Simulated |
|---|---|---|
| 1 | 100.0 | 108.9 |
| 2 | 160.0 | 127.6 |
| 3 | 70.0 | 74.6 |
| 4 | 20.0 | 28.9 |
| 5 | 1.0 | 8.4 |
| 6 | 0.0 | 2.0 |
### Chart: Portugal
| Category | Observed | Simulated |
|---|---|---|
| 1 | 85.0 | 88.0 |
| 2 | 150.0 | 140.6 |
| 3 | 103.0 | 112.5 |
| 4 | 60.0 | 59.8 |
| 5 | 25.0 | 23.9 |
| 6 | 11.0 | 7.6 |
| 7 | 1.0 | 2.0 |
| 8 | 0.0 | 0.5 |
### Chart: Bristol Channel
| Category | Observed | Simulated |
|---|---|---|
| 1 | 113.0 | 120.6 |
| 2 | 165.0 | 129.4 |
| 3 | 55.0 | 68.6 |
| 4 | 15.0 | 24.3 |
| 5 | 3.0 | 6.5 |
| 6 | 0.0 | 1.4 |
### Chart: Mallorca
| Category | Observed | Simulated |
|---|---|---|
| 1 | 266.0 | 254.8 |
| 2 | 210.0 | 205.4 |
| 3 | 131.0 | 151.0 |
| 4 | 133.0 | 92.6 |
| 5 | 32.0 | 46.3 |
| 6 | 8.0 | 19.5 |
| 7 | 0.0 | 7.1 |
### Chart: Crete
| Category | Observed | Simulated |
|---|---|---|
| 1 | 167.0 | 143.6 |
| 2 | 83.0 | 114.7 |
| 3 | 139.0 | 77.0 |
| 4 | 17.0 | 42.4 |
| 5 | 0.0 | 18.6 |
### Chart: Western Channel
| Category | Observed | Simulated |
|---|---|---|
| 1 | 51.0 | 47.5 |
| 2 | 74.0 | 82.8 |
| 3 | 92.0 | 73.3 |
| 4 | 31.0 | 43.5 |
| 5 | 25.0 | 19.4 |
| 6 | 3.0 | 6.8 |
| 7 | 0.0 | 2.0 |
### Chart: Sardinia
| Category | Observed | Simulated |
|---|---|---|
| 1 | 62.0 | 45.7 |
| 2 | 42.0 | 50.7 |
| 3 | 57.0 | 49.6 |
| 4 | 70.0 | 39.2 |
| 5 | 0.0 | 24.5 |
### Chart: Cyprus
| Category | Observed | Simulated |
|---|---|---|
| 1 | 6.0 | 5.7 |
| 2 | 4.0 | 4.4 |
| 3 | 4.0 | 2.8 |
| 4 | 1.0 | 1.4 |
| 5 | 0.0 | 0.5 |
