# Supplementary material for: A tale of two seas: contrasting patterns of population structure in the small-spotted catshark across Europe
Source: R Soc Open Sci. 2014 Nov 12;1(3):140175. doi: 10.1098/rsos.140175 (PMC4448844; doi:10.1098/rsos.140175)
Supplement: SM5 Bayesian Skyline Plots [file rsos140175supp5.docx]

Supplementary Materials 7. Bayesian skyline plots for each sample collection showing the maternal effective population size (mean and 95% confidence interval), back in time (years) since present day. ADR

CRE

SAR

MAL

POR

BRI

WES

IRE

NSE

SCO
